# Crystal structure and Hirshfeld surface analysis of 2-(di­meth­oxy­meth­yl)phenyl (3a*RS*,6*SR*,7*RS*,7a*SR*)-1-oxo-2-phenyl-1,2,3,6,7,7a-hexa­hydro-3a,6-ep­oxy­iso­indole-7-carboxyl­ate

**DOI:** 10.1107/S2056989026010674

**Published:** 2026-10-20

**Authors:** Viktoria S. Maslova, Dayana D. Shadyan, Victor N. Khrustalev, Ibadulla H. Mahmudov, Mehmet Akkurt, Menberu Mengesha Woldemariam, Khudayar I. Hasanov

**Affiliations:** a RUDN University, 6 Miklukho-Maklaya St., Moscow 117198, Russian Federation; b Zelinsky Institute of Organic Chemistry of RAS, Leninsky Prospect 47, Moscow 119991, Russian Federation; c Department of Chemical Engineering, Baku Engineering University, Hasan Aliyev str. 120, AZ0101, Khirdalan, Absheron, Azerbaijan; d Department of Physics, Faculty of Sciences, Erciyes University, 38039 Kayseri, Türkiye; e Department of Physics, Jimma University, Jimma, Ethiopia; f Azerbaijan Medical University, Scientific Research Centre (SRC), A. Kasumzade St. 14, AZ 1022, Baku, Azerbaijan; Universidade de Sâo Paulo, Brazil

**Keywords:** crystal structure, envelope conformation, C—H⋯π inter­actions, Hirshfeld surface analysis

## Abstract

The mol­ecular structure of the title compound, C_24_H_23_NO_6_, exhibits stability due to four intra­molecular C—H⋯O inter­actions that form rings with *S*(6), *S*(9), *S*(5), and *S*(5) motifs. In the 7-oxabi­cyclo­[2.2.1]hept-2-ene unit, the five-membered rings and the pyrrolidine ring all adopt envelope conformations. In the crystal, the mol­ecules are connected by C—H⋯O inter­actions, creating a three-dimensional framework. The mol­ecules are further connected by C—H⋯π inter­actions, forming layers parallel to the (101) plane. Important inter­molecular inter­actions highlighted by Hirshfeld surface analysis are H⋯H (54.6%), C⋯H/H⋯C (21.6%) and O⋯H/H⋯O (21.3%) contacts.

## Abstract 2

In the crystal, the mol­ecules are connected by C—H⋯O inter­actions, creating a three-dimensional framework. The mol­ecules are further connected by C—H⋯π inter­actions, forming layers parallel to the (101) plane.

## 1. Chemical context

3a,6-Ep­oxy­iso­indole carboxyl­ates are of inter­est as rigid polycyclic N,O-containing heterocyclic systems. Their value is related not only to their spatial structure, but also to the possibility of further functionalization: the presence of a carboxyl group makes it possible to obtain esters and introduce various aromatic or biologically significant fragments into the mol­ecule (Varlamov *et al.*, 2004 ▸; Zubkov *et al.*, 2011 ▸). 3a,6-Ep­oxy­iso­indole carb­oxy­lic acids, their esters, and formyl­phenyl derivatives have already been described in the literature, which were then used for the synthesis of azomethines (Dikusar *et al.*, 2024 ▸; Mamedov *et al.*, 2024 ▸). This shows that the ep­oxy­iso­indole framework can be considered as a convenient basis for building more complex mol­ecules, rather than just as a separate synthetic product. Salicyl­aldehyde is also an important and widely used fragment in organic chemistry. Its derivatives readily condense with amines to form Schiff bases, which are actively studied as ligands for metal complexes, fluorescent sensors, and compounds with potential biological activity (Afrin *et al.*, 2023 ▸; Ceramella *et al.*, 2022 ▸; Javadzade *et al.*, 2026 ▸; Li *et al.*, 2025 ▸). Therefore, the combination of the 3a,6-ep­oxy­iso­indole framework with the protected salicyl­aldehyde fragment in the form of acetal is justified: the ep­oxy­iso­indole sets a rigid spatial structure and increases mol­ecular complexity, and the salicyl­aldehyde fragment retains the possibility of further transformations after removing the protection. Thus, the resulting ester can be considered as a hybrid of two synthetically valuable fragments, which expands the range of derivatives of 3a,6-ep­oxy­iso­indoles and may be useful for further preparation of functionalized heterocyclic compounds.
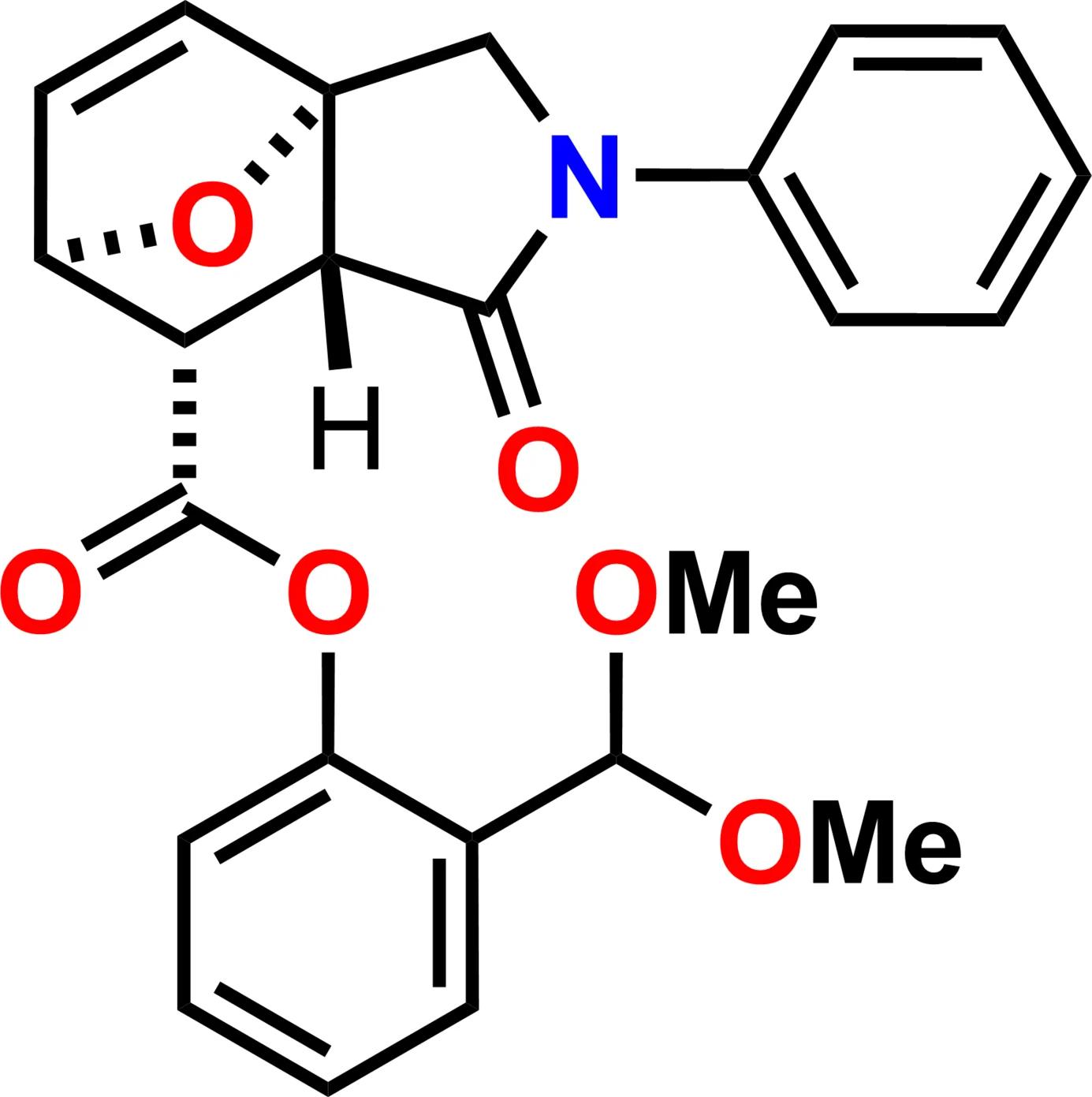


## 2. Structural commentary

The mol­ecular structure of the title compound exhibits stability due to four intra­molecular C—H⋯O inter­actions that form rings with *S*(6), *S*(9), *S*(5), and *S*(5) motifs (Fig. 1 ▸, Table 1 ▸; Bernstein *et al.*, 1995 ▸). The pyrrolidine ring (N2/C1/C7*A*/C3*A*/C3) makes the dihedral angles of 21.14 (11) and 21.56 (10)°, respectively, with the phenyl ring (C8–C13) and the benzene ring (C15–C20), while these rings subtend a dihedral angle of 13.69 (10)° with each other. In the 7-oxabi­cyclo­[2.2.1]hept-2-ene unit of the ten-membered fused 1,2,3,6,7,7a-hexa­hydro-3a,6-ep­oxy­iso­indole ring system, the (O8/C3*A*/C4/C5/C6) and (O8/C3*A*/C7*A*/C7/C6) rings adopt envelope conformations with the puckering parameters (Cremer & Pople, 1975 ▸) *Q*(2) = 0.511 (2) Å, φ(2) = 181.3 (3)° and *Q*(2) = 0.599 (2) Å, φ(2) = 3.6 (2)°, respectively, and atom O8 as the flap in both cases. The pyrrolidine ring (N2/C1/C7*A*/C3*A*/C3) adopts an envelope conformation on atom C3*A* as the flap with puckering parameters *Q*(2) = 0.317 (2) Å, φ(2) = 282.4 (4)°.

## 3. Supra­molecular features and Hirshfeld surface analysis

There were no classical hydrogen bonds in the crystal. The mol­ecules are linked by C—H⋯O inter­actions, forming a three-dimensional network (Figs. 2 ▸, 3 ▸ and 4 ▸, Table 1 ▸). The mol­ecules are further linked by C—H⋯π inter­actions, forming layers that are oriented with the (10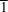) plane (Figs. 5 ▸ and 6 ▸, Table 1 ▸). No π–π inter­actions were observed.

*CrystalExplorer 21* (Spackman *et al.*, 2021 ▸) was used to construct Hirshfeld surfaces and generate the related two-dimensional fingerprint plots to illustrate the inter­molecular inter­actions for the mol­ecules of the title compound. The *d*_norm_ mappings were conducted in the range −0.2256 to +1.3633 a.u. Bright-red circles on the *d*_norm_ surfaces (Fig. 7 ▸) represent O—H⋯O and C—H⋯O inter­action zones (Table 1 ▸ and 2 ▸).

Two-dimensional fingerprint plots of the title compound together with their percentage contributions are shown in Fig. 8 ▸. The crystal packing is dominated by H⋯H contacts, representing van der Waals inter­actions (54.6% contribution to the overall surface), followed by C⋯H/H⋯C and O⋯H/H⋯O inter­actions, which contribute to 21.6% and 21.3%, respectively. The other contacts [N⋯H/H⋯N (1.3%), O⋯C/C⋯O (0.6%), C⋯C (0.5%) and N⋯C/C⋯N (0.1%)] only make a minor contribution to the crystal packing.

## 4. Database survey

A search of the Cambridge Structural Database (CSD version 6.01, updated February 2026; Groom *et al.*, 2016 ▸) for the 1,2,3,6,7,7a-hexa­hydro-3a,6-ep­oxy­iso­indole ring system returned five matches. These are CSD refcodes ERIVIL (Temel *et al.*, 2011 ▸), OMUTAU (Mertsalov *et al.*, 2021 ▸), UPAQEI (Koşar *et al.*, 2011 ▸), TIJMIK (Demircan *et al.*, 2013 ▸), and YAXCIL (Temel *et al.*, 2012 ▸).

ERIVIL and OMUTAU crystallize in the triclinic space group *P*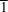 while UPAQEI, TIJMIK and YAXCIL crystallize in the monoclinic space group *P*2_1_/*c*,

In ERIVIL, the two tetra­hydro­furan rings adopt envelope conformations, the pyrrolidine ring adopts a half-chair conformation and the six-membered ring is in a boat conformation. In the crystal, weak inter­molecular C—H⋯O hydrogen bonds link the mol­ecules into 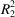
(8) and 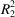
(14) rings along the *b-*axis direction.

In OMUTAU, the tetra­hydro­furan rings of both mol­ecules (*A* and *B*) in the asymmetric unit, adopt envelope conformations, the pyrrolidine rings adopt a twisted-envelope conformation and the six-membered ring is in a boat conformation. In the crystal, strong inter­molecular O—H⋯O hydrogen bonds and weak inter­molecular C—H⋯O contacts link the mol­ecules, forming a three-dimensional network. In addition weak π–π stacking inter­actions are observed of the *A* mol­ecules.

In UPAQEI, the boat form of the six-membered ring is almost symmetrical with respect to the ep­oxy bridge. The two five-membered rings generated by the ep­oxy bridge of the six-membered ring adopt envelope conformations, whereas the N-containing five-membered ring adopts a twisted conformation. In the crystal, mol­ecules are linked by C—H⋯O hydrogen bonds.

In TIJMIK, the tetra­hydro­furan ring adopts an envelope conformation. The pyrrolidine ring adopts an envelope conformation. In the crystal, two types of C—H⋯O hydrogen bonds generate 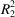
(20) and 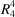
(26) rings, with adjacent rings running parallel to *ac* plane. Further C—H⋯O hydrogen bonds form a *C*(6) chain, linking the mol­ecules in the *b-*axis direction.

In YAXCIL, the six-membered ring has a boat conformation. The two five-membered rings with the bridging O atom adopt envelope conformations, whereas the N-containing five-membered ring adopts a twisted conformation. In the crystal, C—H⋯O hydrogen bonds link the mol­ecules into a three-dimensional network.

## 5. Synthesis and crystallization

A solution of the 2-formyl­phenyl (3a*RS*,6*SR*,7*RS*,7a*SR*)-1-oxo-2-phenyl-1,2,3,6,7,7a-hexa­hydro-3a,6-ep­oxy­iso­indole-7-carboxyl­ate (0.1 g, 0.27 mmol, 1.0 equiv) in methanol (3 mL) was treated with methano­lic hydrogen chloride (1.25 *M* solution in MeOH, 22 µL, 0.027 mmol, 0.1 equiv). The reaction mixture was stirred at room temperature for 7 days (TLC control, hexa­ne–EtOAc, 1:2). After completion of the reaction, the reaction mixture was poured into water (50 mL). The precipitate was filtered off, washed with water (3 × 5 mL), dried under vacuum, and air-dried to afford the corresponding acetal as a colorless powder, yield 25%, 28 mg (0.07 mmol), m.p. 437–440 K. Single crystals of the 2-(di­meth­oxy­meth­yl)phenyl (3a*RS*,6*SR*,7*RS*,7a*SR*)-1-oxo-2-phenyl-1,2,3,6,7,7a-hexa­hydro-3a,6-ep­oxy­iso­indole-7-carboxyl­ate were grown from the 1:1 *v*/*v* mixture of a CHCl_3_/EtOH at 281 K. ^1^H NMR (700.2 MHz, CDCl_3_), (*J*, Hz) 7.61–7.60 (*m*, 3 H, H-Ar), 7.48 (*d*, *J* = 8.1 Hz, 1 H, H-Ar), 7.41–7.36 (*m*, 3 H, H-Ar), 7.25 (*t*, *J* = 7.6 Hz, 1 H, H-Ar), 7.16 (*t*, *J* = 8.1 Hz, 1 H, H-Ar), 6.65 (*d*, *J* = 5.7 Hz, 1 H, H-4), 6.59 (*dd*, *J* = 5.7, 1.4 Hz, 1 H, H-5), 5.65 (*s*, 1 H, C*H*(OCH_3_)_2_), 5.43 (*d*, *J* = 1.4 Hz, 1 H, H-6), 4.50 and 4.24 (*two d*, *J* = 11.4 Hz, 2 H, H-3), 3.30 (*s*, 3 H, C*H*_3_), 3.26 (*s*, 3 H, C*H*_3_), 3.15 and 3.05 (*two d*, *J* = 9.1 Hz, 2 H, H-7,7a). ^13^C NMR (176.1 MHz, CDCl_3_) δ 170.0, 169.9, 148.8, 139.1, 137.3, 135.5, 129.8, 129.6, 128.9 (2C), 127.8, 125.5, 125.0, 123.4, 120.5 (2C), 98.7, 87.8, 81.9, 52.8, 52.4, 52.2, 50.2, 45.8. MS (ESI) *m*/*z*: [*M*]^+^ 422.

## 6. Refinement

Crystal data, data collection and structure refinement details are summarized in Table 3 ▸. H atoms were placed in idealized positions and allowed to ride on their parent atoms, with C—H = 0.95, 0.98, 0.99 and 1.00 Å for aromatic, methyl, methyl­ene and methine H respectively, and *U*_iso_(H) = 1.5*U*_eq_(C) for methyl H and *U*_iso_(H) = 1.2*U*_eq_(C) for all other H atoms.

## Supplementary Material

Crystal structure: contains datablock(s) I. DOI: 10.1107/S2056989026010674/ex2108sup1.cif

Structure factors: contains datablock(s) I. DOI: 10.1107/S2056989026010674/ex2108Isup2.hkl

Supporting information file. DOI: 10.1107/S2056989026010674/ex2108Isup3.cml

CCDC reference: 2596512

Additional supporting information:  crystallographic information; 3D view; checkCIF report

## Figures and Tables

**Figure 1:**
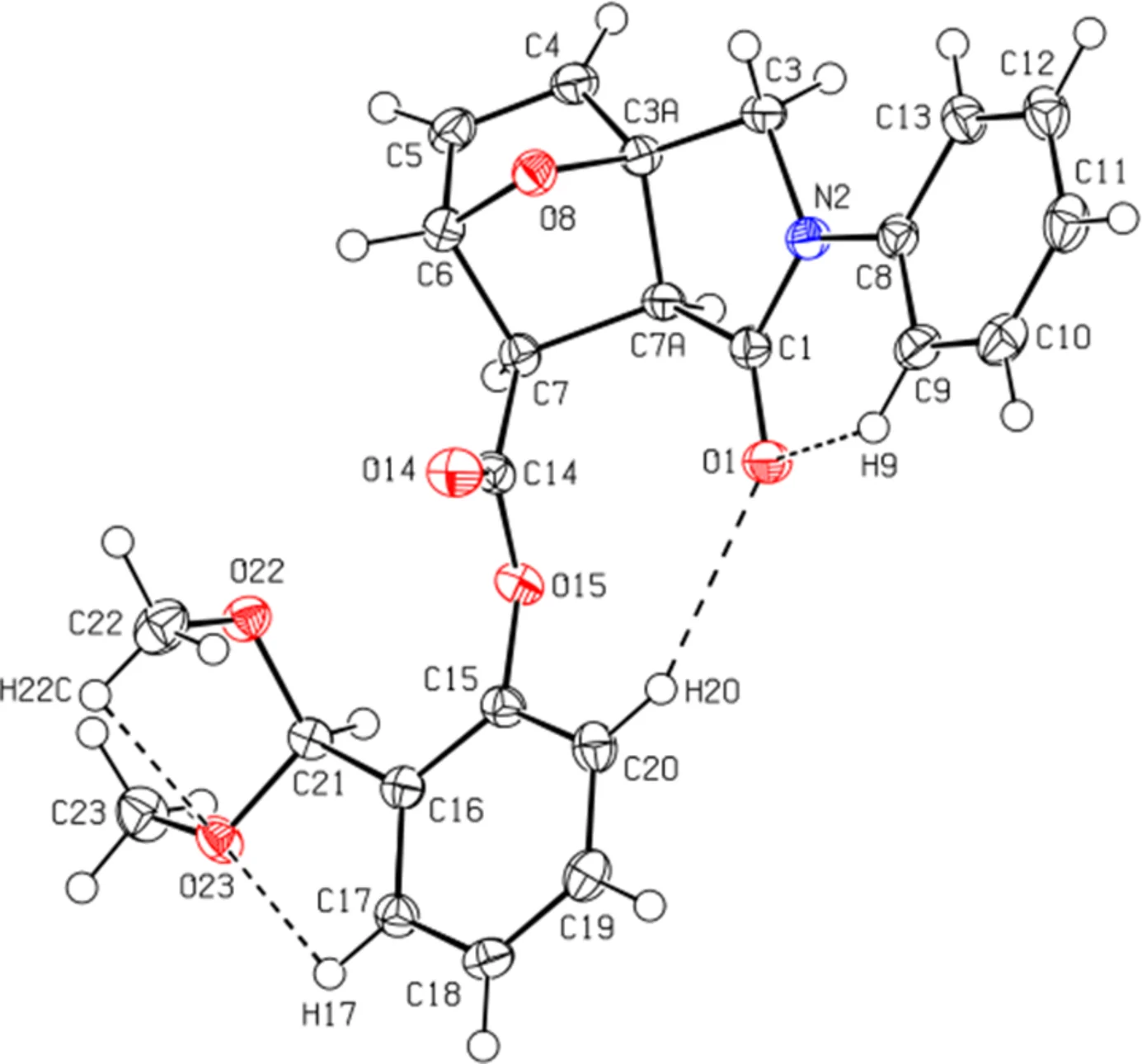
Mol­ecular structure of the title compound showing the atom labeling and ring system with displacement ellipsoids at 50% probability level. The intra­molecular C9—H9⋯O1, C20—H20⋯O1, C17—H17⋯O23 and C22—H22*C*⋯O23 inter­actions, shown as dashed lines, enclose *S*(6), *S*(9), *S*(5) and *S*(5) motifs, respectively.

**Figure 2:**
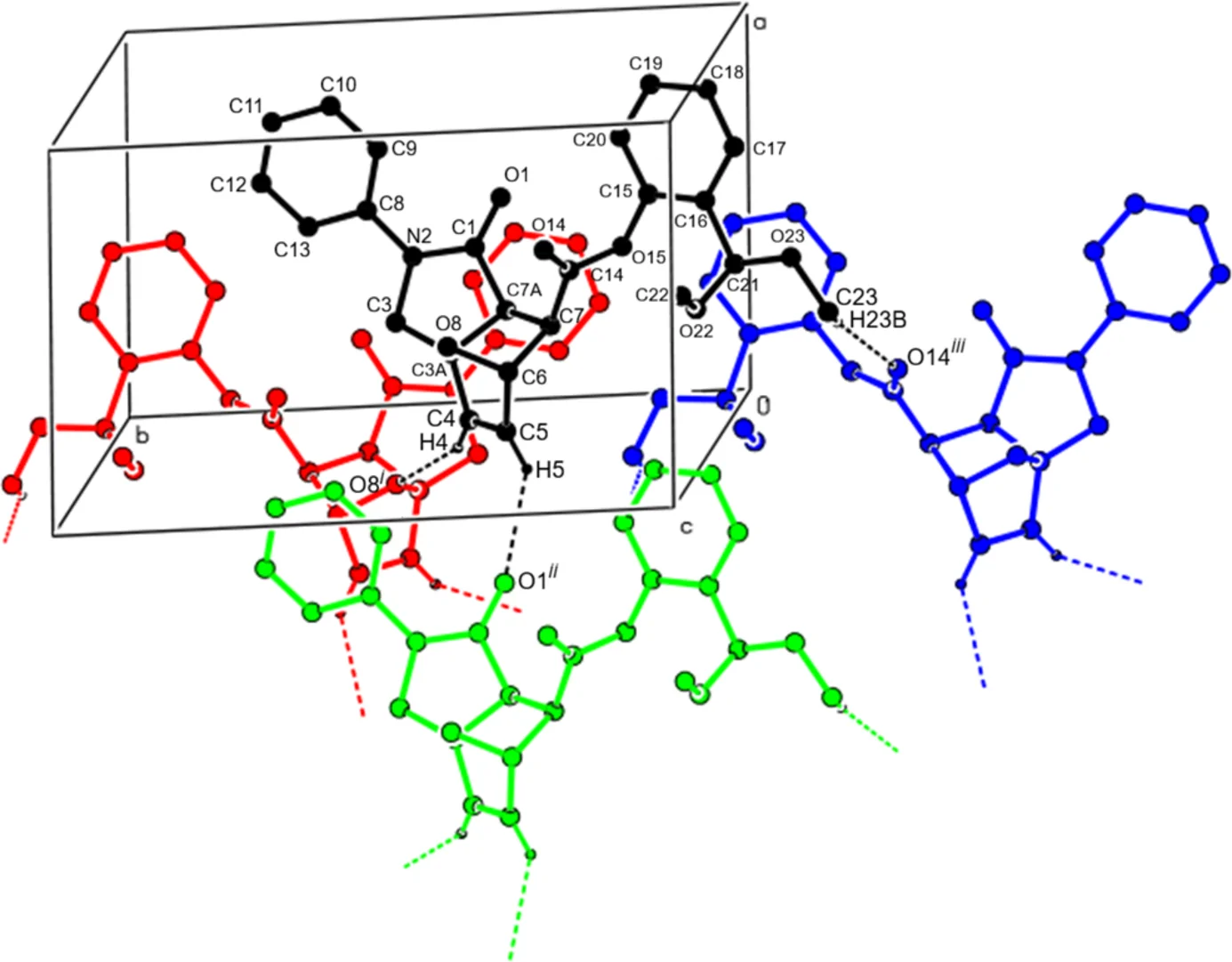
Partial packing view showing the C4—H4⋯O8^i^, C5—H5⋯O1^ii^ and C23—H23*B*⋯O14^iii^ inter­actions in the unit cell. Symmetry codes: (i) *x* − 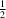, −*y* + 1, *z* − 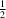; (ii) *x* − 1, *y*, *z*; (iii) *x* − 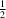, −*y*, *z* − 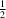.

**Figure 3:**
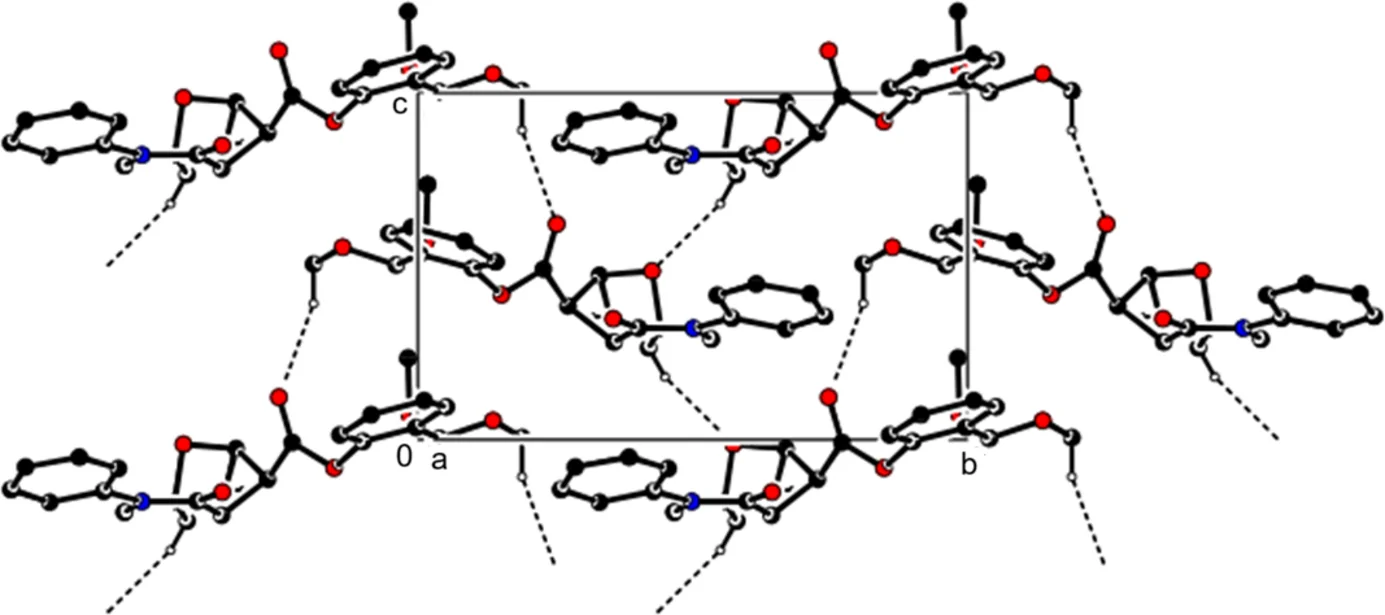
A view of the C—H⋯O inter­actions along the *a*-axis direction.

**Figure 4:**
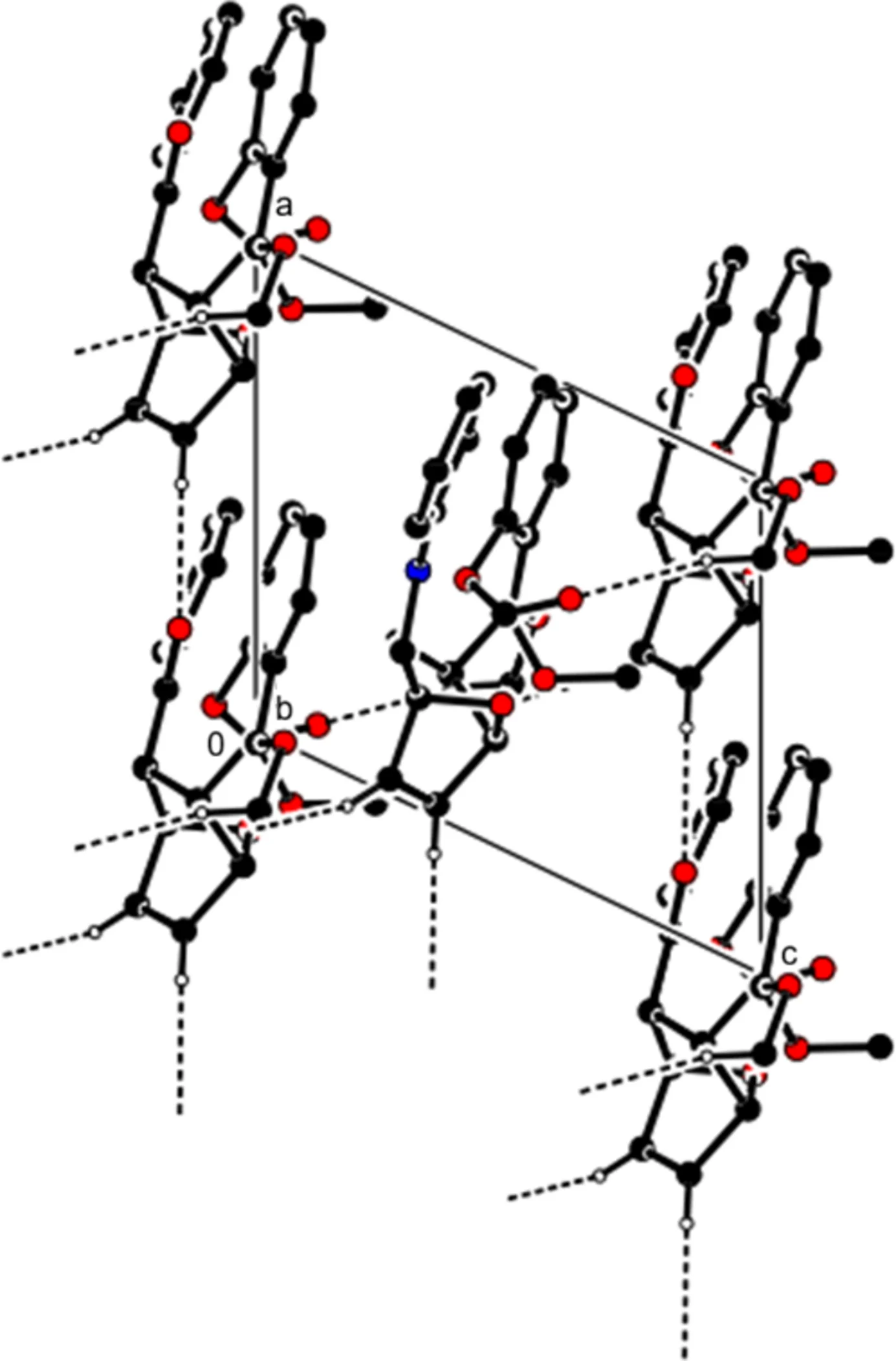
A view of the C—H⋯O inter­actions along the *b*-axis direction.

**Figure 5:**
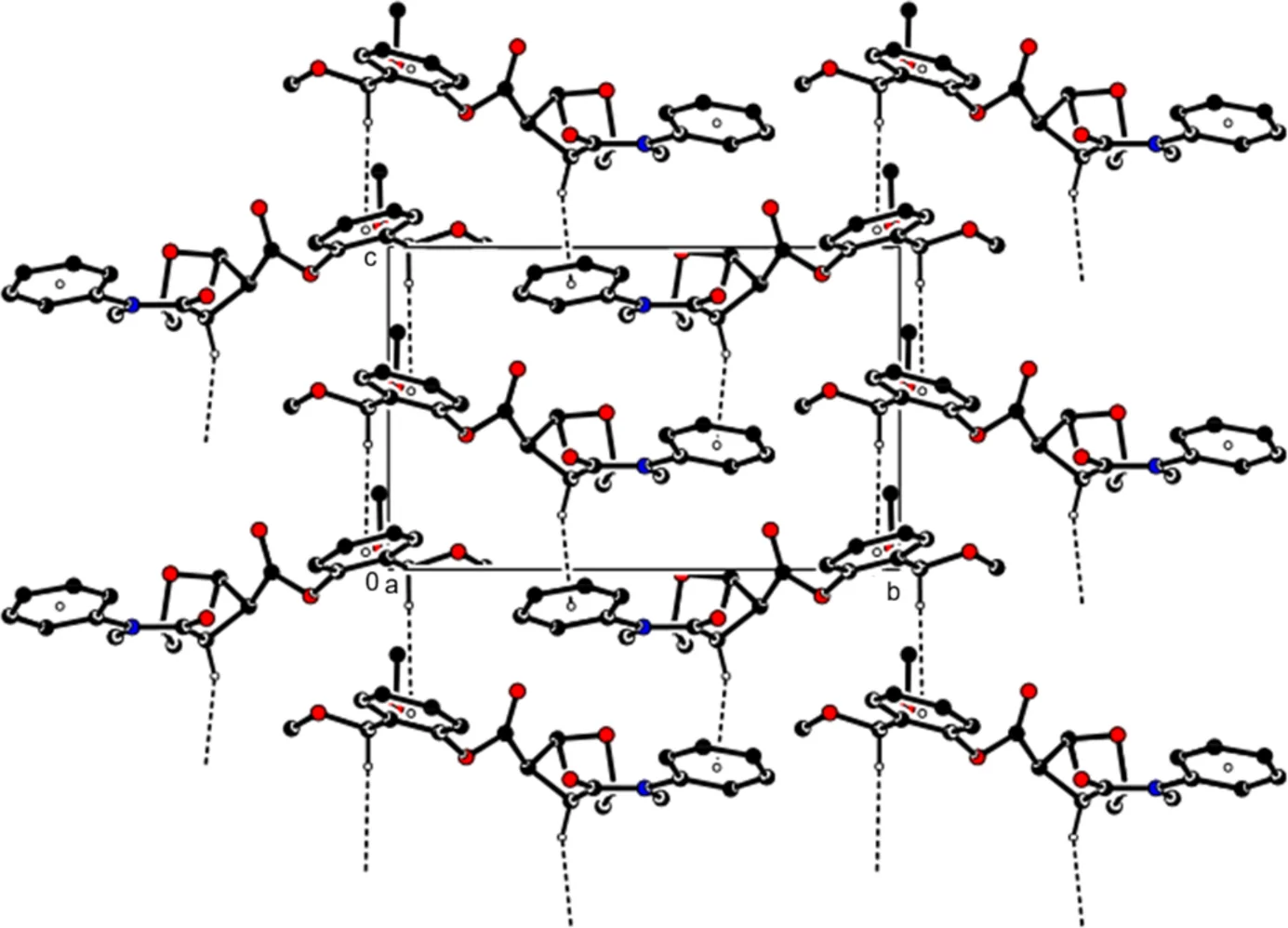
A view of the C—H⋯π inter­actions along the *a*-axis direction.

**Figure 6:**
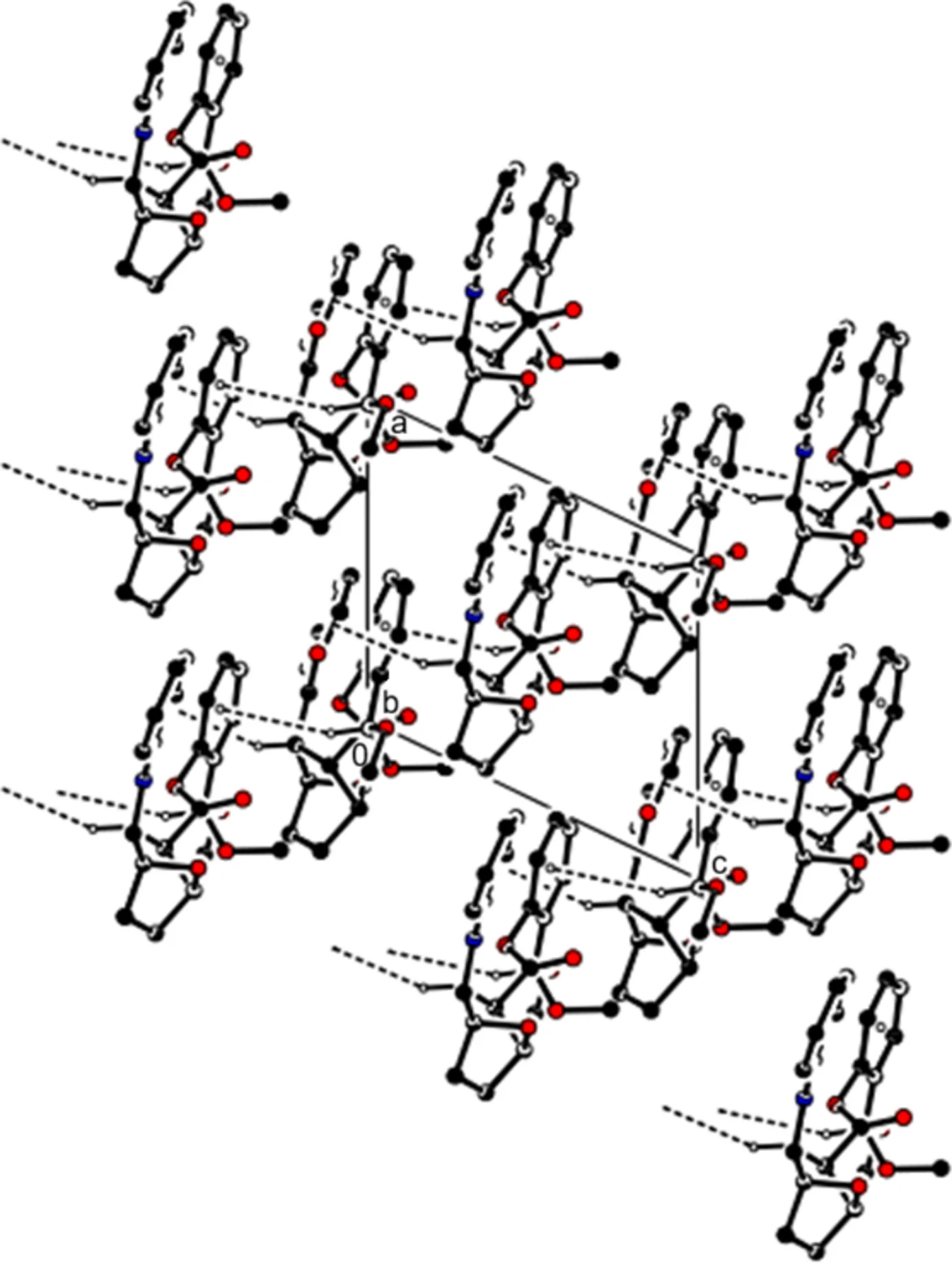
A view of the C—H⋯π inter­actions along the *b*-axis direction.

**Figure 7:**
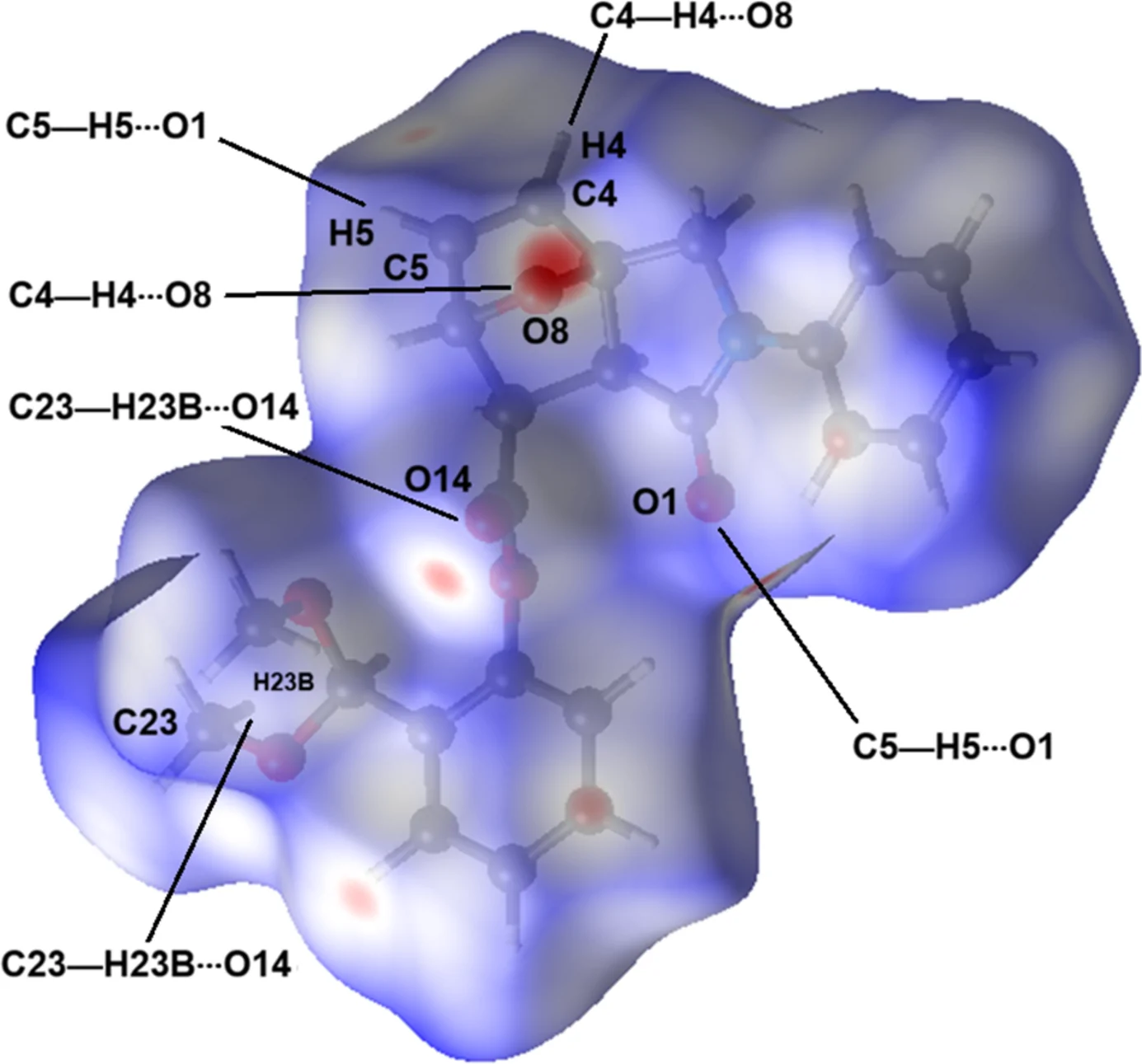
View of the three-dimensional Hirshfeld surface of the title compound mapped over *d*_norm_ in the range −0.2256 to 1.3633 a.u.

**Figure 8:**
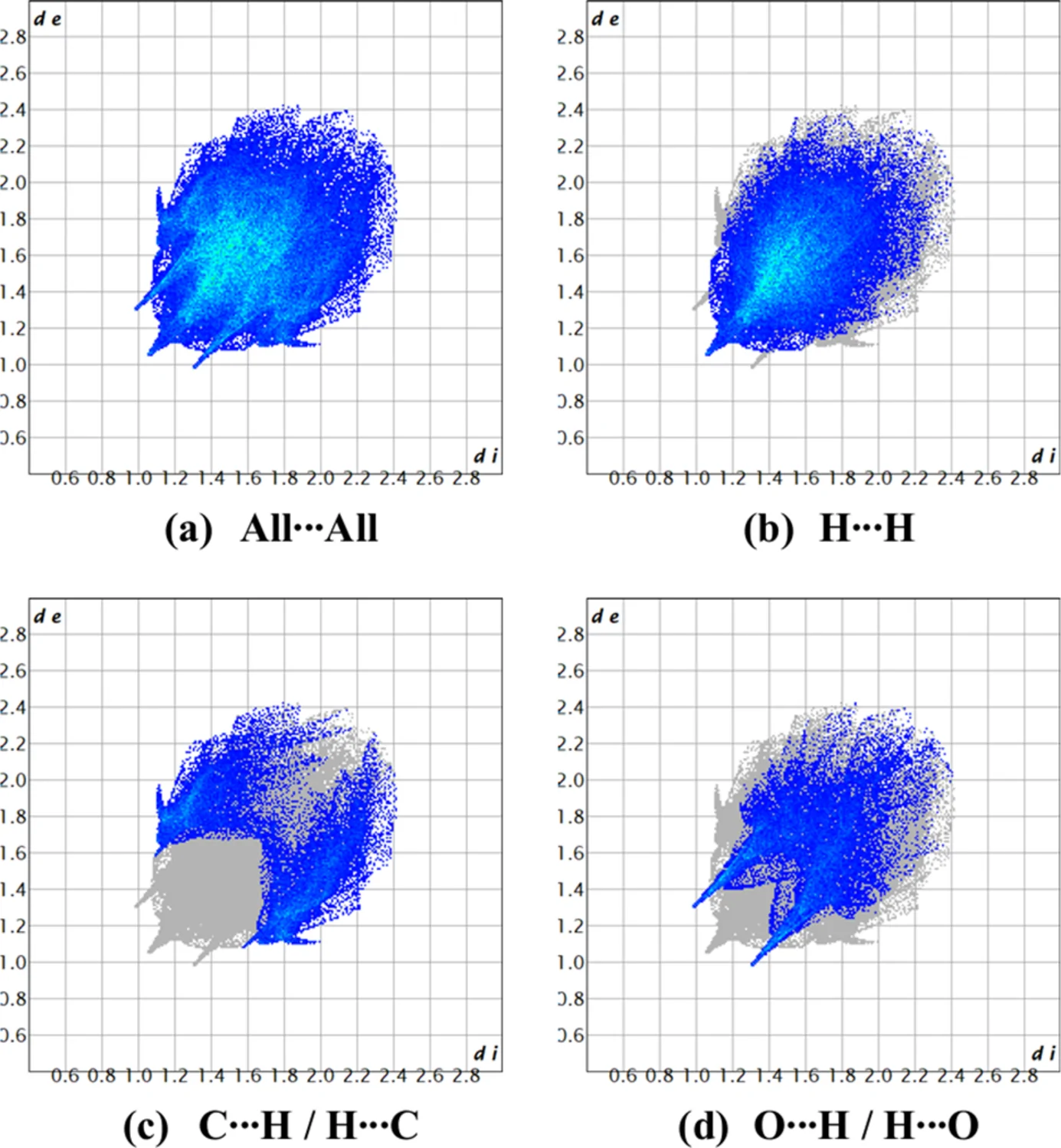
The two-dimensional fingerprint plots of the title compound, showing (*a*) all inter­actions, and those delineated into (*b*) H⋯H, (*c*) C⋯H/H⋯C, (*d*) O⋯H/H⋯O inter­actions. The *d*_i_ and *d*_e_ values are the closest inter­nal and external distances (in Å) from given points on the Hirshfeld surface.

**Table 1:** Hydrogen-bond geometry (Å, °) *Cg*5 and *Cg*6 are the centroids of the C8—C13 phenyl ring and the C15–C20 benzene ring, respectively.

*D*—H⋯*A*	*D*—H	H⋯*A*	*D*⋯*A*	*D*—H⋯*A*
C4—H4⋯O8^i^	0.95	2.42	3.314 (3)	156
C5—H5⋯O1^ii^	0.95	2.53	3.314 (3)	140
C9—H9⋯O1	0.95	2.24	2.863 (3)	123
C17—H17⋯O23	0.95	2.36	2.702 (3)	101
C20—H20⋯O1	0.95	2.58	3.387 (3)	143
C22—H22*C*⋯O23	0.98	2.49	2.823 (3)	100
C23—H23*B*⋯O14^iii^	0.98	2.57	3.492 (3)	157
C7*A*—H7*A*⋯*Cg*5^i^	1.00	2.68	3.564 (2)	147
C21—H21⋯*Cg*6^iii^	1.00	2.96	3.912 (2)	159

Symmetry codes: (i) 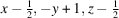; (ii) 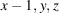; (iii) 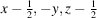.

**Table 2:** Summary of short inter­atomic contacts (Å)

Contact	Distance	Symmetry operation
H20⋯H5	2.34	1 + *x*, *y*, *z*
O8⋯H4	2.42	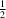 + *x*, 1 − *y*, 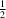 + *z*
H22*B*⋯H21	2.45	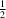 + *x*, −*y*, 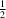 + *z*
C12⋯H6	2.87	− 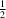 + *x*, 1 − *y*, − 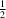 + *z*
H12⋯C17	2.95	*x*, 1 + *y*, *z*
C19⋯H22*C*	2.92	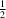 + *x*, −*y*, − 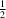 + *z*
H23*C*⋯H11	2.57	−1 + *x*, −1 + *y*, *z*

**Table 3:** Experimental details

Crystal data	
Chemical formula	C_24_H_23_NO_6_
*M*_r_	421.43
Crystal system, space group	Monoclinic, *Pn*
Temperature (K)	100
*a*, *b*, *c* (Å)	8.4620 (1), 13.6810 (17), 9.5690 (13)
β (°)	115.731 (6)
*V* (Å^3^)	997.94 (19)
*Z*	2
Radiation type	Synchrotron, λ = 0.75170 Å
μ (mm^−1^)	0.11
Crystal size (mm)	0.12 × 0.08 × 0.06

Data collection	
Diffractometer	Rayonix SX165 CCD
Absorption correction	Multi-scan (*SCALA*; Evans, 2006 ▸)
*T*_min_, *T*_max_	0.963, 0.987
No. of measured, independent and observed [*I* > 2σ(*I*)] reflections	11428, 5347, 4834
*R*_int_	0.034
(sin θ/λ)_max_ (Å^−1^)	0.688

Refinement	
*R*[*F*^2^ > 2σ(*F*^2^)], *wR*(*F*^2^), *S*	0.035, 0.084, 1.04
No. of reflections	5347
No. of parameters	282
No. of restraints	2
H-atom treatment	H-atom parameters constrained
Δρ_max_, Δρ_min_ (e Å^−3^)	0.18, −0.20
Absolute structure	Flack *x* determined using 2152 quotients [(*I*^+^)−(*I*^−^)]/[(*I*^+^)+(*I*^−^)] (Parsons *et al.*, 2013 ▸)
Absolute structure parameter	0.2 (3)

Computer programs: *Marccd* (Doyle, 2011 ▸), *iMosflm* (Battye *et al.*, 2011 ▸), *SHELXT* (Sheldrick, 2015*a* ▸), *SHELXL* (Sheldrick, 2015*b* ▸), *ORTEP-3 for Windows* (Farrugia, 2012 ▸) and *PLATON* (Spek, 2020 ▸).

## supplementary crystallographic information

### 2-(Dimethoxymethyl)phenyl (3aRS,6SR,7RS,7aSR)-1-oxo-2-phenyl-1,2,3,6,7,7a-hexahydro-3a,6-epoxyisoindole-7-carboxylate Crystal data

**Table:**

C_24_H_23_NO_6_	*F*(000) = 444
*M**_r_* = 421.43	*D*_x_ = 1.403 Mg m^−^^3^
Monoclinic, *P**n*	Synchrotron radiation, λ = 0.75170 Å
*a* = 8.4620 (1) Å	Cell parameters from 1000 reflections
*b* = 13.6810 (17) Å	θ = 3.0–30.0°
*c* = 9.5690 (13) Å	µ = 0.11 mm^−^^1^
β = 115.731 (6)°	*T* = 100 K
*V* = 997.94 (19) Å^3^	Prism, colourless
*Z* = 2	0.12 × 0.08 × 0.06 mm

### 2-(Dimethoxymethyl)phenyl (3aRS,6SR,7RS,7aSR)-1-oxo-2-phenyl-1,2,3,6,7,7a-hexahydro-3a,6-epoxyisoindole-7-carboxylate Data collection

**Table:**

Rayonix SX165 CCD diffractometer	4834 reflections with *I* > 2σ(*I*)
/f scan	*R*_int_ = 0.034
Absorption correction: multi-scan (*SCALA*; Evans, 2006)	θ_max_ = 31.1°, θ_min_ = 3.0°
*T*_min_ = 0.963, *T*_max_ = 0.987	*h* = −11→11
11428 measured reflections	*k* = −18→18
5347 independent reflections	*l* = −13→13

### 2-(Dimethoxymethyl)phenyl (3aRS,6SR,7RS,7aSR)-1-oxo-2-phenyl-1,2,3,6,7,7a-hexahydro-3a,6-epoxyisoindole-7-carboxylate Refinement

**Table:**

Refinement on *F*^2^	Secondary atom site location: difference Fourier map
Least-squares matrix: full	Hydrogen site location: inferred from neighbouring sites
*R*[*F*^2^ > 2σ(*F*^2^)] = 0.035	H-atom parameters constrained
*wR*(*F*^2^) = 0.084	*w* = 1/[σ^2^(*F*_o_^2^) + (0.0432*P*)^2^ + 0.1076*P*] where *P* = (*F*_o_^2^ + 2*F*_c_^2^)/3
*S* = 1.04	(Δ/σ)_max_ < 0.001
5347 reflections	Δρ_max_ = 0.18 e Å^−^^3^
282 parameters	Δρ_min_ = −0.20 e Å^−^^3^
2 restraints	Absolute structure: Flack *x* determined using 2152 quotients [(*I*^+^)-(*I*^-^)]/[(*I*^+^)+(*I*^-^)] (Parsons *et al.*, 2013)
Primary atom site location: difference Fourier map	Absolute structure parameter: 0.2 (3)

### 2-(Dimethoxymethyl)phenyl (3aRS,6SR,7RS,7aSR)-1-oxo-2-phenyl-1,2,3,6,7,7a-hexahydro-3a,6-epoxyisoindole-7-carboxylate Special details

**Table:**

Geometry. All esds (except the esd in the dihedral angle between two l.s. planes) are estimated using the full covariance matrix. The cell esds are taken into account individually in the estimation of esds in distances, angles and torsion angles; correlations between esds in cell parameters are only used when they are defined by crystal symmetry. An approximate (isotropic) treatment of cell esds is used for estimating esds involving l.s. planes.

### 2-(Dimethoxymethyl)phenyl (3aRS,6SR,7RS,7aSR)-1-oxo-2-phenyl-1,2,3,6,7,7a-hexahydro-3a,6-epoxyisoindole-7-carboxylate Fractional atomic coordinates and isotropic or equivalent isotropic displacement parameters (Å^2^)

**Table:**

	*x*	*y*	*z*	*U*_iso_*/*U*_eq_	
C1	0.4961 (3)	0.40137 (15)	0.3223 (2)	0.0190 (4)	
O1	0.6301 (2)	0.35560 (11)	0.34868 (19)	0.0247 (3)	
N2	0.4799 (2)	0.50154 (13)	0.3215 (2)	0.0201 (3)	
C3	0.3012 (3)	0.53354 (15)	0.2908 (3)	0.0212 (4)	
H3A	0.234768	0.555430	0.182043	0.025*	
H3B	0.304050	0.587019	0.361521	0.025*	
C3A	0.2241 (3)	0.44210 (15)	0.3220 (2)	0.0190 (4)	
C4	0.0320 (3)	0.41977 (16)	0.2651 (2)	0.0224 (4)	
H4	−0.063836	0.449515	0.181188	0.027*	
C5	0.0260 (3)	0.34939 (17)	0.3580 (3)	0.0246 (4)	
H5	−0.075822	0.317177	0.352920	0.029*	
C6	0.2148 (3)	0.33061 (16)	0.4743 (2)	0.0216 (4)	
H6	0.229673	0.300789	0.574637	0.026*	
C7	0.3029 (3)	0.27320 (15)	0.3857 (2)	0.0186 (4)	
H7	0.221508	0.221351	0.319527	0.022*	
C7A	0.3171 (3)	0.35687 (15)	0.2817 (2)	0.0181 (4)	
H7A	0.255702	0.339462	0.169199	0.022*	
O8	0.2907 (2)	0.42620 (11)	0.48675 (16)	0.0214 (3)	
C8	0.6140 (3)	0.57109 (15)	0.3512 (2)	0.0197 (4)	
C9	0.7912 (3)	0.54460 (16)	0.4169 (2)	0.0235 (4)	
H9	0.824033	0.477938	0.438949	0.028*	
C10	0.9183 (3)	0.61563 (18)	0.4496 (3)	0.0269 (5)	
H10	1.038297	0.596990	0.492347	0.032*	
C11	0.8743 (3)	0.71356 (18)	0.4212 (3)	0.0277 (5)	
H11	0.962897	0.761937	0.445983	0.033*	
C12	0.6989 (3)	0.73968 (17)	0.3563 (3)	0.0266 (5)	
H12	0.667261	0.806645	0.336615	0.032*	
C13	0.5684 (3)	0.66946 (16)	0.3194 (2)	0.0234 (4)	
H13	0.448485	0.688281	0.272662	0.028*	
C14	0.4759 (3)	0.22845 (14)	0.4920 (2)	0.0191 (4)	
O14	0.5702 (2)	0.25257 (12)	0.62243 (17)	0.0266 (3)	
O15	0.50935 (19)	0.15215 (11)	0.41812 (16)	0.0206 (3)	
C15	0.6653 (3)	0.09970 (15)	0.4948 (2)	0.0191 (4)	
C16	0.6535 (3)	0.00321 (15)	0.5349 (2)	0.0187 (4)	
C17	0.8069 (3)	−0.05195 (15)	0.5946 (2)	0.0203 (4)	
H17	0.803329	−0.118128	0.623109	0.024*	
C18	0.9650 (3)	−0.01146 (16)	0.6129 (2)	0.0226 (4)	
H18	1.068604	−0.050075	0.653582	0.027*	
C19	0.9727 (3)	0.08477 (17)	0.5722 (2)	0.0243 (4)	
H19	1.081235	0.111944	0.584366	0.029*	
C20	0.8222 (3)	0.14171 (16)	0.5137 (2)	0.0235 (4)	
H20	0.826585	0.208218	0.487071	0.028*	
C21	0.4759 (3)	−0.03941 (15)	0.5039 (2)	0.0189 (4)	
H21	0.402850	−0.039989	0.389011	0.023*	
O22	0.3843 (2)	0.01482 (12)	0.57138 (17)	0.0231 (3)	
C22	0.4688 (3)	0.0178 (2)	0.7361 (3)	0.0304 (5)	
H22A	0.395144	0.053485	0.774374	0.046*	
H22B	0.582221	0.050938	0.770393	0.046*	
H22C	0.487505	−0.049026	0.777041	0.046*	
O23	0.5021 (2)	−0.13678 (11)	0.55526 (18)	0.0230 (3)	
C23	0.3417 (3)	−0.18961 (18)	0.5065 (3)	0.0287 (5)	
H23A	0.366870	−0.257094	0.544044	0.043*	
H23B	0.280366	−0.189553	0.392951	0.043*	
H23C	0.267661	−0.158497	0.548995	0.043*	

### 2-(Dimethoxymethyl)phenyl (3aRS,6SR,7RS,7aSR)-1-oxo-2-phenyl-1,2,3,6,7,7a-hexahydro-3a,6-epoxyisoindole-7-carboxylate Atomic displacement parameters (Å^2^)

**Table:**

	*U*^11^	*U*^22^	*U*^33^	*U*^12^	*U*^13^	*U*^23^
C1	0.0200 (9)	0.0184 (9)	0.0198 (9)	−0.0005 (8)	0.0097 (7)	0.0002 (7)
O1	0.0216 (7)	0.0192 (7)	0.0356 (8)	0.0010 (6)	0.0148 (6)	0.0017 (6)
N2	0.0191 (8)	0.0178 (8)	0.0251 (8)	−0.0011 (7)	0.0110 (7)	−0.0009 (6)
C3	0.0201 (9)	0.0183 (10)	0.0269 (10)	0.0031 (8)	0.0119 (8)	0.0019 (8)
C3A	0.0194 (9)	0.0197 (9)	0.0185 (9)	0.0024 (8)	0.0090 (7)	0.0010 (7)
C4	0.0202 (10)	0.0211 (10)	0.0269 (10)	0.0020 (8)	0.0113 (8)	−0.0002 (8)
C5	0.0210 (10)	0.0259 (11)	0.0306 (11)	0.0003 (9)	0.0148 (8)	−0.0001 (8)
C6	0.0241 (10)	0.0207 (10)	0.0236 (9)	0.0003 (8)	0.0135 (8)	0.0009 (7)
C7	0.0186 (9)	0.0182 (9)	0.0188 (8)	−0.0010 (7)	0.0079 (7)	0.0007 (7)
C7A	0.0172 (9)	0.0169 (9)	0.0204 (8)	0.0023 (7)	0.0084 (7)	0.0007 (7)
O8	0.0243 (7)	0.0212 (7)	0.0200 (7)	0.0002 (6)	0.0108 (6)	−0.0007 (5)
C8	0.0228 (10)	0.0188 (9)	0.0199 (9)	−0.0015 (8)	0.0115 (8)	−0.0014 (7)
C9	0.0243 (11)	0.0238 (11)	0.0262 (10)	0.0004 (9)	0.0145 (9)	−0.0020 (8)
C10	0.0242 (10)	0.0296 (12)	0.0308 (11)	−0.0048 (9)	0.0156 (9)	−0.0056 (8)
C11	0.0330 (12)	0.0259 (11)	0.0294 (11)	−0.0103 (10)	0.0183 (9)	−0.0055 (9)
C12	0.0356 (12)	0.0207 (10)	0.0259 (11)	−0.0045 (9)	0.0156 (9)	−0.0012 (8)
C13	0.0280 (10)	0.0204 (10)	0.0227 (9)	−0.0015 (9)	0.0118 (8)	0.0005 (8)
C14	0.0204 (9)	0.0154 (9)	0.0213 (9)	−0.0009 (7)	0.0088 (7)	0.0009 (7)
O14	0.0265 (8)	0.0283 (8)	0.0209 (7)	0.0030 (7)	0.0065 (6)	−0.0030 (6)
O15	0.0202 (7)	0.0178 (7)	0.0202 (6)	0.0016 (6)	0.0053 (5)	0.0002 (5)
C15	0.0192 (9)	0.0195 (9)	0.0168 (8)	0.0017 (8)	0.0062 (7)	0.0000 (7)
C16	0.0203 (9)	0.0201 (9)	0.0146 (8)	0.0001 (8)	0.0065 (7)	−0.0021 (7)
C17	0.0221 (9)	0.0199 (9)	0.0181 (9)	0.0014 (8)	0.0080 (7)	0.0004 (7)
C18	0.0209 (10)	0.0263 (10)	0.0197 (9)	0.0049 (8)	0.0079 (7)	−0.0021 (7)
C19	0.0215 (10)	0.0293 (11)	0.0230 (9)	−0.0045 (9)	0.0106 (8)	−0.0037 (8)
C20	0.0254 (11)	0.0222 (10)	0.0228 (9)	−0.0036 (9)	0.0106 (8)	−0.0003 (8)
C21	0.0187 (9)	0.0178 (9)	0.0190 (8)	0.0005 (7)	0.0070 (7)	−0.0002 (7)
O22	0.0199 (7)	0.0266 (8)	0.0224 (7)	0.0021 (6)	0.0090 (6)	−0.0002 (6)
C22	0.0289 (11)	0.0405 (13)	0.0224 (10)	0.0014 (10)	0.0116 (9)	−0.0040 (9)
O23	0.0211 (7)	0.0182 (7)	0.0272 (7)	−0.0014 (6)	0.0082 (6)	0.0033 (6)
C23	0.0262 (11)	0.0242 (11)	0.0332 (12)	−0.0061 (9)	0.0104 (9)	−0.0007 (9)

### 2-(Dimethoxymethyl)phenyl (3aRS,6SR,7RS,7aSR)-1-oxo-2-phenyl-1,2,3,6,7,7a-hexahydro-3a,6-epoxyisoindole-7-carboxylate Geometric parameters (Å, º)

**Table:**

C1—O1	1.222 (3)	C11—H11	0.9500
C1—N2	1.377 (3)	C12—C13	1.388 (3)
C1—C7A	1.518 (3)	C12—H12	0.9500
N2—C8	1.412 (3)	C13—H13	0.9500
N2—C3	1.476 (3)	C14—O14	1.200 (3)
C3—C3A	1.499 (3)	C14—O15	1.357 (2)
C3—H3A	0.9900	O15—C15	1.398 (2)
C3—H3B	0.9900	C15—C20	1.385 (3)
C3A—O8	1.442 (2)	C15—C16	1.390 (3)
C3A—C4	1.505 (3)	C16—C17	1.392 (3)
C3A—C7A	1.546 (3)	C16—C21	1.516 (3)
C4—C5	1.326 (3)	C17—C18	1.387 (3)
C4—H4	0.9500	C17—H17	0.9500
C5—C6	1.521 (3)	C18—C19	1.382 (3)
C5—H5	0.9500	C18—H18	0.9500
C6—O8	1.439 (3)	C19—C20	1.387 (3)
C6—C7	1.562 (3)	C19—H19	0.9500
C6—H6	1.0000	C20—H20	0.9500
C7—C14	1.503 (3)	C21—O23	1.404 (2)
C7—C7A	1.555 (3)	C21—O22	1.416 (2)
C7—H7	1.0000	C21—H21	1.0000
C7A—H7A	1.0000	O22—C22	1.421 (3)
C8—C13	1.396 (3)	C22—H22A	0.9800
C8—C9	1.399 (3)	C22—H22B	0.9800
C9—C10	1.381 (3)	C22—H22C	0.9800
C9—H9	0.9500	O23—C23	1.425 (3)
C10—C11	1.386 (4)	C23—H23A	0.9800
C10—H10	0.9500	C23—H23B	0.9800
C11—C12	1.384 (4)	C23—H23C	0.9800
			
O1—C1—N2	126.32 (19)	C12—C11—C10	118.9 (2)
O1—C1—C7A	125.50 (19)	C12—C11—H11	120.6
N2—C1—C7A	108.17 (17)	C10—C11—H11	120.6
C1—N2—C8	126.88 (18)	C11—C12—C13	121.0 (2)
C1—N2—C3	112.78 (17)	C11—C12—H12	119.5
C8—N2—C3	120.30 (18)	C13—C12—H12	119.5
N2—C3—C3A	102.07 (16)	C12—C13—C8	119.9 (2)
N2—C3—H3A	111.4	C12—C13—H13	120.1
C3A—C3—H3A	111.4	C8—C13—H13	120.1
N2—C3—H3B	111.4	O14—C14—O15	123.97 (19)
C3A—C3—H3B	111.4	O14—C14—C7	127.16 (19)
H3A—C3—H3B	109.2	O15—C14—C7	108.84 (16)
O8—C3A—C3	110.27 (16)	C14—O15—C15	118.94 (15)
O8—C3A—C4	102.26 (16)	C20—C15—C16	122.51 (19)
C3—C3A—C4	126.31 (18)	C20—C15—O15	119.22 (19)
O8—C3A—C7A	99.17 (15)	C16—C15—O15	117.93 (18)
C3—C3A—C7A	105.52 (16)	C15—C16—C17	117.55 (19)
C4—C3A—C7A	110.09 (17)	C15—C16—C21	119.63 (18)
C5—C4—C3A	104.76 (18)	C17—C16—C21	122.72 (18)
C5—C4—H4	127.6	C18—C17—C16	120.80 (19)
C3A—C4—H4	127.6	C18—C17—H17	119.6
C4—C5—C6	106.21 (19)	C16—C17—H17	119.6
C4—C5—H5	126.9	C19—C18—C17	120.4 (2)
C6—C5—H5	126.9	C19—C18—H18	119.8
O8—C6—C5	101.32 (17)	C17—C18—H18	119.8
O8—C6—C7	101.62 (16)	C18—C19—C20	120.1 (2)
C5—C6—C7	106.58 (16)	C18—C19—H19	120.0
O8—C6—H6	115.2	C20—C19—H19	120.0
C5—C6—H6	115.2	C15—C20—C19	118.7 (2)
C7—C6—H6	115.2	C15—C20—H20	120.7
C14—C7—C7A	114.19 (16)	C19—C20—H20	120.7
C14—C7—C6	113.07 (16)	O23—C21—O22	111.82 (17)
C7A—C7—C6	99.47 (16)	O23—C21—C16	107.70 (16)
C14—C7—H7	109.9	O22—C21—C16	113.67 (17)
C7A—C7—H7	109.9	O23—C21—H21	107.8
C6—C7—H7	109.9	O22—C21—H21	107.8
C1—C7A—C3A	101.11 (16)	C16—C21—H21	107.8
C1—C7A—C7	118.48 (16)	C21—O22—C22	114.17 (17)
C3A—C7A—C7	101.94 (15)	O22—C22—H22A	109.5
C1—C7A—H7A	111.4	O22—C22—H22B	109.5
C3A—C7A—H7A	111.4	H22A—C22—H22B	109.5
C7—C7A—H7A	111.4	O22—C22—H22C	109.5
C6—O8—C3A	95.62 (14)	H22A—C22—H22C	109.5
C13—C8—C9	119.2 (2)	H22B—C22—H22C	109.5
C13—C8—N2	118.94 (19)	C21—O23—C23	112.55 (16)
C9—C8—N2	121.80 (19)	O23—C23—H23A	109.5
C10—C9—C8	119.8 (2)	O23—C23—H23B	109.5
C10—C9—H9	120.1	H23A—C23—H23B	109.5
C8—C9—H9	120.1	O23—C23—H23C	109.5
C9—C10—C11	121.3 (2)	H23A—C23—H23C	109.5
C9—C10—H10	119.4	H23B—C23—H23C	109.5
C11—C10—H10	119.4		
			
O1—C1—N2—C8	−0.2 (3)	C3—N2—C8—C13	−13.3 (3)
C7A—C1—N2—C8	−179.18 (18)	C1—N2—C8—C9	−13.4 (3)
O1—C1—N2—C3	−178.0 (2)	C3—N2—C8—C9	164.23 (19)
C7A—C1—N2—C3	3.1 (2)	C13—C8—C9—C10	0.1 (3)
C1—N2—C3—C3A	17.1 (2)	N2—C8—C9—C10	−177.4 (2)
C8—N2—C3—C3A	−160.84 (17)	C8—C9—C10—C11	1.2 (3)
N2—C3—C3A—O8	76.45 (19)	C9—C10—C11—C12	−1.2 (3)
N2—C3—C3A—C4	−160.02 (18)	C10—C11—C12—C13	−0.2 (3)
N2—C3—C3A—C7A	−29.7 (2)	C11—C12—C13—C8	1.4 (3)
O8—C3A—C4—C5	−33.7 (2)	C9—C8—C13—C12	−1.4 (3)
C3—C3A—C4—C5	−160.6 (2)	N2—C8—C13—C12	176.19 (19)
C7A—C3A—C4—C5	70.9 (2)	C7A—C7—C14—O14	91.2 (3)
C3A—C4—C5—C6	1.4 (2)	C6—C7—C14—O14	−21.6 (3)
C4—C5—C6—O8	31.3 (2)	C7A—C7—C14—O15	−90.62 (19)
C4—C5—C6—C7	−74.6 (2)	C6—C7—C14—O15	156.59 (17)
O8—C6—C7—C14	87.89 (19)	O14—C14—O15—C15	−2.3 (3)
C5—C6—C7—C14	−166.43 (17)	C7—C14—O15—C15	179.40 (17)
O8—C6—C7—C7A	−33.62 (18)	C14—O15—C15—C20	−73.4 (2)
C5—C6—C7—C7A	72.07 (19)	C14—O15—C15—C16	113.2 (2)
O1—C1—C7A—C3A	159.9 (2)	C20—C15—C16—C17	−0.7 (3)
N2—C1—C7A—C3A	−21.1 (2)	O15—C15—C16—C17	172.60 (17)
O1—C1—C7A—C7	49.6 (3)	C20—C15—C16—C21	−177.05 (18)
N2—C1—C7A—C7	−131.42 (18)	O15—C15—C16—C21	−3.8 (3)
O8—C3A—C7A—C1	−82.73 (16)	C15—C16—C17—C18	0.0 (3)
C3—C3A—C7A—C1	31.42 (19)	C21—C16—C17—C18	176.26 (18)
C4—C3A—C7A—C1	170.53 (16)	C16—C17—C18—C19	0.1 (3)
O8—C3A—C7A—C7	39.83 (18)	C17—C18—C19—C20	0.4 (3)
C3—C3A—C7A—C7	153.98 (15)	C16—C15—C20—C19	1.2 (3)
C4—C3A—C7A—C7	−66.91 (19)	O15—C15—C20—C19	−171.98 (18)
C14—C7—C7A—C1	−14.7 (3)	C18—C19—C20—C15	−1.1 (3)
C6—C7—C7A—C1	106.01 (19)	C15—C16—C21—O23	−179.92 (17)
C14—C7—C7A—C3A	−124.49 (18)	C17—C16—C21—O23	3.9 (3)
C6—C7—C7A—C3A	−3.79 (19)	C15—C16—C21—O22	−55.5 (2)
C5—C6—O8—C3A	−49.64 (17)	C17—C16—C21—O22	128.4 (2)
C7—C6—O8—C3A	60.13 (17)	O23—C21—O22—C22	58.6 (2)
C3—C3A—O8—C6	−172.14 (17)	C16—C21—O22—C22	−63.6 (2)
C4—C3A—O8—C6	51.28 (17)	O22—C21—O23—C23	65.2 (2)
C7A—C3A—O8—C6	−61.75 (17)	C16—C21—O23—C23	−169.22 (17)
C1—N2—C8—C13	169.08 (19)		

### 2-(Dimethoxymethyl)phenyl (3aRS,6SR,7RS,7aSR)-1-oxo-2-phenyl-1,2,3,6,7,7a-hexahydro-3a,6-epoxyisoindole-7-carboxylate Hydrogen-bond geometry (Å, º)

*Cg*5 and *Cg*6 are the centroids of the C8—C23 phenyl ring and the C15–C20 benzene ring, respectively.

**Table:**

*D*—H···*A*	*D*—H	H···*A*	*D*···*A*	*D*—H···*A*
C4—H4···O8^i^	0.95	2.42	3.314 (3)	156
C5—H5···O1^ii^	0.95	2.53	3.314 (3)	140
C9—H9···O1	0.95	2.24	2.863 (3)	123
C17—H17···O23	0.95	2.36	2.702 (3)	101
C20—H20···O1	0.95	2.58	3.387 (3)	143
C22—H22*C*···O23	0.98	2.49	2.823 (3)	100
C23—H23*B*···O14^iii^	0.98	2.57	3.492 (3)	157
C7*A*—H7*A*···Cg5^i^	1.00	2.68	3.564 (2)	147
C21—H21···Cg6^iii^	1.00	2.96	3.912 (2)	159

Symmetry codes: (i) *x*−1/2, −*y*+1, *z*−1/2; (ii) *x*−1, *y*, *z*; (iii) *x*−1/2, −*y*, *z*−1/2.
